# Medical Cannabis as an Opiate Alternative: A Prospective Observational Cohort Study

**DOI:** 10.7759/cureus.107248

**Published:** 2026-04-17

**Authors:** Franklin E Caldera, Zachary Caldera, Frances Shofer, Benjamin Abramoff, David Lenrow, Stephen Hampton, Adrian Popescu, Timothy Dillingham

**Affiliations:** 1 Physical Medicine and Rehabilitation, University of Pennsylvania Perelman School of Medicine, Philadelphia, USA; 2 Emergency Medicine, University of Pennsylvania Perelman School of Medicine, Philadelphia, USA

**Keywords:** cannabis, chronic pain, medical marijuana, medications, opioids

## Abstract

Background

Opioid use for chronic pain has contributed to an epidemic of overdoses and deaths in the United States. Some clinical studies suggest that medical cannabis may help alleviate pain and improve quality of life. However, cost can be a barrier to patients accessing medical cannabis. This is the first prospective observational study evaluating medical cannabis as an alternative to opioids in a setting where cost was removed as a major barrier.

Methods

Twenty-nine patients were recruited from a university-based outpatient chronic pain clinic. Each patient underwent monthly pain assessments using the Numeric Pain Rating Scale (NRS). Daily opioid use, measured in morphine milligram equivalents (MMEs), was recorded and monitored over five months. Pain-related quality of life was assessed using the SF-36 Health Survey at baseline, and again at two and five months.

Results

Daily opioid use decreased from baseline to one month (from 46.8 MMEs to 16.2 MMEs, a 65% reduction), and this reduction was maintained through five months. The mean NRS score decreased by two points from baseline to one month (from 7.03 to 5.07; p < 0.0001), and this improvement was sustained at five months. The SF-36 Physical Functioning score increased from 15.3 at baseline to 21.4 at one month and was maintained at five months (p < 0.03 for both comparisons).

Conclusion

These results suggest that medical cannabis may be a useful adjunct therapy for reducing opioid use, relieving chronic pain, and improving health-related quality of life.

## Introduction

The ongoing opioid epidemic in the United States represents one of the most significant public health crises in the nation’s history, and effective interventions have been challenging to implement [1,2]. Approximately 806,000 people died from opioid overdoses between 1999 and 2023 [3,4]. One of the root causes of the epidemic was the overprescription of opioid medications during the 1990s, driven in part by advocacy from organizations such as the American Pain Society to treat pain as the “fifth vital sign,” as well as efforts by hospital administrators to increase patient satisfaction [5]. Due to its role in the development of the opioid crisis, the American Medical Association voted in 2016 to discontinue the use of pain as a vital sign. Clinicians have since been encouraged to adopt more comprehensive approaches to pain assessment and management [6].

Opioid medications exert their effects by binding to mu-opioid receptors in the central nervous system, including the spinal cord and brain regions involved in pain perception and reward, resulting in both analgesia and euphoria [7]. Prolonged use can lead to tolerance and physiological dependence. Evidence suggests that opioids provide only modest short-term improvements in pain and function, while posing a significantly higher risk of misuse and addiction compared to non-opioid therapies [8-11].

Another contributing factor to the opioid epidemic has been the increasing stigma associated with nonsteroidal anti-inflammatory drugs (NSAIDs). Although NSAIDs are associated with mild increases in the risk of renal and cardiovascular adverse events, their benefits have often been overlooked. Concerns about medico-legal risk may have contributed to the reduction in prescribing of these effective medications. Recent evidence suggests that NSAIDs are safe and may be more effective than opioids for chronic joint pain and low back pain [12].

One potential alternative or adjunct to opioid therapy is medical cannabis, which has been proposed as a safer option for the treatment of chronic pain. The cannabis plant has been used medicinally for thousands of years [13,14]. Its primary active compounds, Δ9-tetrahydrocannabinol (THC) and cannabidiol (CBD), both influence brain function [15,16].

THC produces a range of acute effects, including lightheadedness, euphoria, tachycardia, and impaired concentration. In contrast, CBD may influence mood and cognition but does not produce intoxicating effects, even at high doses. When co-administered, CBD may modulate the effects of THC depending on the route of administration and relative doses [17]. Both THC and CBD have demonstrated analgesic and potential therapeutic properties for pain.

Cannabis is currently classified as a Schedule I substance under federal law in the United States, which has historically limited the ability to conduct large, rigorous clinical trials evaluating its efficacy for pain management. Despite this, several studies, including randomized placebo-controlled trials, have examined its use in chronic pain.

A survey-based study found that 97% of patients using medical cannabis alongside opioids agreed or strongly agreed that they were able to reduce their opioid dose, and 71% reported that cannabis provided similar pain relief [18]. A prospective study of 129 patients with chronic musculoskeletal pain reported that 51.1% strongly agreed and 42.6% agreed that cannabis improved their primary symptom [19]. Price et al. conducted a systematic review and concluded that cannabis may be effective for back pain with an acceptable side effect profile [20].

It is also important to consider potential risks associated with cannabis use. Its use has increased for both recreational and medical purposes, and some evidence suggests an association with neurocognitive impairment and reduced brain volume. Early use may carry a risk of long-term deficits [21].

Based on available evidence, medical cannabis appears to be a potentially safe and effective option for pain relief. However, because it is classified as a Schedule I substance under federal law, it is not covered by insurance, creating a financial barrier for many patients. We hypothesize that improving access to medical cannabis will enable a subset of patients, particularly those for whom cost is a major barrier, to reduce or discontinue opioid use while maintaining adequate pain control. Given the lack of prospective studies evaluating outcomes when this barrier is removed, this study addresses an important gap in the existing literature.

## Materials and methods

This was a pre-post cohort study conducted in an outpatient chronic pain clinic at the Hospital of the University of Pennsylvania, Department of Physical Medicine and Rehabilitation. Inclusion criteria were as follows: (1) current use of opioid medication for chronic pain; (2) interest in reducing or discontinuing opioid use; (3) prior inability to taper opioid medications despite alternative pharmacologic and interventional treatments; (4) willingness to use medical cannabis for pain management, with cost identified as a prior barrier; and (5) a qualifying medical condition under the Pennsylvania Department of Health Medical Marijuana Program. Patients were excluded if they had a history of schizophrenia or acute psychiatric illness (e.g., bipolar disorder or major depression), held a firearm permit, or were employed in a position that prohibited medical cannabis use. Patients were also excluded if they were unable to obtain a medical cannabis card from the Pennsylvania Department of Health following initial evaluation.

After providing informed consent, eligibility for medical cannabis was confirmed by a physician certified to evaluate patients for the program. Patients then registered with the Pennsylvania Department of Health Medical Marijuana Program. With guidance from a medical cannabis pharmacist at a state-approved dispensary, patients selected from available cost-subsidized cannabis products. Each patient was subsequently started on an individualized opioid tapering plan.

Prior to initiating medical cannabis, each patient underwent a urine drug screen, a pain assessment using the Numeric Pain Rating Scale (NRS) [22], and a pain-related quality-of-life assessment using the RAND Short Form-36 (SF-36) Health Survey [23], a 36-item questionnaire developed as part of the Medical Outcomes Study. These assessments were repeated at two and five months after initiation of medical cannabis. In addition, patients had monthly clinical visits with a physician over a five-month period. During these visits, pain levels (measured using the NRS) and daily opioid use (measured in morphine milligram equivalents, MMEs) were recorded. Patients were also monitored for side effects, tolerance, and changes in symptoms. The primary outcomes were changes in pain scores and opioid use over time. Secondary outcomes included side effects and changes in health-related quality of life as measured by the SF-36 Health Survey. 

Statistical analysis

Demographic data are presented as frequencies and percentages, means with standard deviations, or medians with interquartile ranges, as appropriate. To evaluate changes in opioid consumption and pain levels over time, linear mixed-effects models were used, with time (baseline and months 1-5) treated as a repeated measure and age included as a covariate. Due to non-normal distribution, opioid consumption data were log-transformed using log10(MME + 1). Results are reported as mean NRS scores and the antilog-transformed values of (MME - 1), each with 95% CIs. A separate linear mixed-effects model was used to analyze SF-36 scores at three time points (baseline, two months, and five months). To account for multiple comparisons, post hoc pairwise comparisons were performed using the Tukey-Kramer method. All analyses were conducted using SAS statistical software (version 9.4; SAS Inc, Cary, NC, USA). Figures were generated using GraphPad Prism (version 10.6.1; GraphPad Software, San Diego, CA, USA). The study protocol was approved by the University of Pennsylvania Institutional Review Board (IRB #856134).

## Results

Thirty-nine patients met the inclusion criteria and were screened for the study. Of these, 10 were excluded due to psychiatric illness (e.g., a history of acute psychiatric illness or schizophrenia), leaving a final sample of 29 patients. The cohort included 19 (65.5%) women and 25 (86%) African American participants, with a mean age of 63 years (range 29-80). The most common chronic pain diagnosis was lumbar degenerative disc disease (n = 10, 34%), followed by lumbar radiculopathy (n = 9, 31%). The median duration of opioid use was 11 years (range 3-14 years). Notably, all included patients reported cost as the primary barrier to using medical cannabis prior to this study.

**Figure 1:**
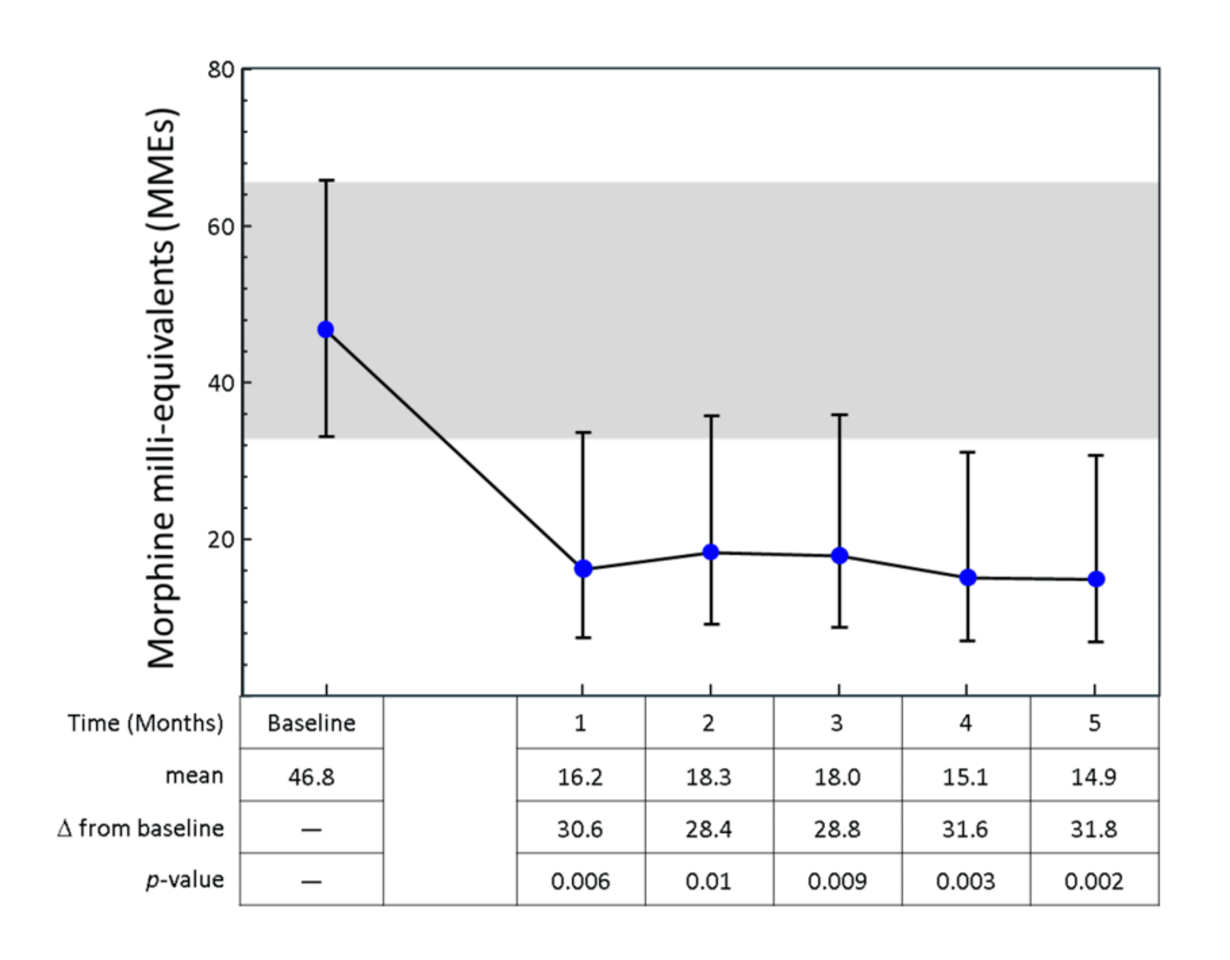
Opioid consumption over time. Error bars represent the mean antilog of opioids consumed (MME - 1) with 95% CIs. The p-values are derived from pairwise Tukey-Kramer tests of change in MMEs from baseline for each time point.

Mean daily opioid consumption decreased from a baseline of 46.8 MMEs/day (95% CI: 37.3-58.6) to 16.2 MMEs/day at one month and remained low throughout the five-month follow-up period (range 14.9-18.3 MMEs/day; p < 0.01) (Figure 1).

**Figure 2:**
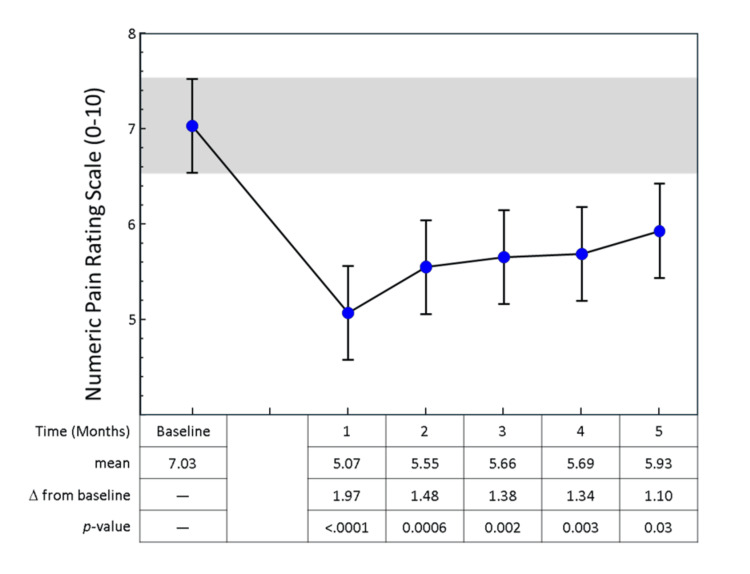
Numeric Pain Rating Scale (NRS) over time. Error bars represent the mean NRS with 95% CIs. The p-values are derived from pairwise Tukey-Kramer tests of change in NRS from baseline for each time point.

Seven patients (24%) were able to completely discontinue opioid therapy by the end of the study, five of whom achieved this by the second month. Pain levels also decreased over time. The baseline NRS score (mean = 7.0, 95% CI: 6.3-7.7) decreased by an average of 1.1-2.0 points during follow-up (p < 0.03 for all comparisons from baseline) (Figure 2).

The SF-36 Physical Functioning subscale score (with higher scores indicating better health-related functioning) increased from 15.3% at baseline to 21.4% at two months and 21.6% at five months (p < 0.03 for both comparisons) (Table 1). The Health Change domain also increased by 17% at two months and 11% at five months; however, these changes were not statistically significant. All other SF-36 domains remained unchanged.

**Table 1:** SF-36 components over time. Values represent the mean score at each time point. The p-values represent overall model significance.

SF-36 component	Baseline	Two months	Five months	p-value
Physical functioning	15.3	21.4	21.6	0.013
Health Change	38.8	56.0	50.0	0.058
Vitality (energy/fatigue)	32.1	38.1	35.3	0.177
Bodily pain	25.5	29.2	28.8	0.410
General health	41.7	41.0	39.1	0.662
Social functioning	44.4	46.9	49.1	0.682
Emotional well-being	34.5	43.7	40.2	0.698

## Discussion

To our knowledge, this is the first prospective study evaluating whether medical cannabis can be used as an alternative to opioids in patients with chronic pain for whom cost has been a primary barrier to access.

In this study, there was a statistically significant reduction in mean pain scores that was sustained over the five-month study period. There was also a reduction in mean opioid consumption of approximately 32 MMEs per day, which was similarly sustained throughout the follow-up. In addition, seven patients were able to discontinue opioid therapy completely during the study.

These findings are consistent with prior research demonstrating the potential benefits of cannabis in pain management. Takakuwa et al. found that cannabis was effective as an adjunct to opioid therapy for low back pain in a long-term observational study [24]. Takakuwa and Sulak also reported that cannabis may serve as a substitute for prescription opioids and is associated with reduced opioid use among patients with chronic pain [25]. Additional studies have reported benefits in other chronic pain conditions, including fibromyalgia and spinal cord injury [26-28].

Patients in this study consistently identified cost as a major barrier to initiating medical cannabis. This is particularly relevant, given that cannabis remains classified as a Schedule I substance under the US federal law, which precludes insurance coverage even for medical use. The findings of this study add to the growing body of literature supporting the safety profile and potential therapeutic role of cannabis. These data may help inform future considerations regarding reclassification, which could reduce financial barriers to access and help destigmatize its use for pain management and other medical indications.

Despite several strengths, including the prospective design and use of validated outcome measures, this study has important limitations. Pain scores were self-reported and therefore subjective. The sample size was small and derived from a single clinical site, and there was no control group. Additionally, patients self-titrated medical cannabis products, resulting in variability in dosing and frequency of use.

## Conclusions

Opioid misuse related to chronic pain treatment remains a significant public health challenge in the United States. Opioids are associated with serious adverse events, including death, particularly at higher dosages (≥90 MMEs/day) or when used in combination with benzodiazepines. In contrast, medical cannabis has not been associated with mortality from overdose. When used under appropriate medical supervision, medical cannabis may represent an effective adjunctive strategy for reducing opioid use among patients receiving long-term opioid therapy. However, substantial barriers to access and limitations in rigorous clinical research remain. Although cannabis has historically been characterized as a potential “gateway drug,” it may also serve as a harm-reduction tool for some patients seeking to reduce reliance on higher-risk opioid medications. Further research with longer follow-up periods and standardized cannabis formulations and dosing is needed to better define the role of medical cannabis as a sustainable alternative or adjunct to opioid therapy.
